# Meta-analysis of Xiaoyao formula as an adjuvant therapy for treating postpartum depression

**DOI:** 10.3389/fpsyt.2025.1558505

**Published:** 2025-03-24

**Authors:** Jing Liu, Anna Rong, Fei Wang

**Affiliations:** Department of Obstetrics and Gynecology, The Affiliated Hospital of Inner Mongolia Medical University, Hohhot, China

**Keywords:** traditional Chinese medicine, Xiaoyao powder, Xiaoyao pill, postpartum depression, randomized controlled trials, meta-analysis

## Abstract

**Background:**

Xiaoyao Powder/Pill, as a classical Chinese herbal formula, has been widely used for treating depression. This meta-analysis aims to evaluate the effectiveness of Xiaoyao formula (XYF) as an adjuvant therapy for treating postpartum depression.

**Methods:**

We searched studies indexed in international databases (Cochrane Library, Embase, and PubMed) and Chinese databases (SinoMed, CNKI, and Wanfang). Randomized controlled trials (RCTs) assessing the efficacy of XYF in combination with Western therapy compared to Western therapy alone for treating postpartum depression were eligible. The total response rate was defined as a reduction of more than 25% in depression scores, while clinical recovery was defined as a reduction of more than 75% in depression scores.

**Results:**

Our analysis included ten RCTs comprising 810 women. The combination of XYF and Western therapy led to a significant improvement in the total response rate (risk ratios [RR] 1.17; confidence intervals [CI] 1.08-1.26) and the clinical recovery rate (RR 1.56; 95% CI 1.27-1.91) compared to Western therapy alone. Additionally, XYF as an adjuvant therapy also significantly decreased Hamilton Depression Scale scores (standard mean difference [SMD] -1.69; 95% CI -2.37 to -1.01).

**Conclusions:**

Adjuvant treatment with XYF can effectively alleviate depression in postpartum women. However, further well-designed RCTs are necessary to validate these findings, as the current evidence remains uncertain.

## Introduction

Postpartum depression are serious mental illnesses that can affect women after giving birth. Around 17% of healthy delivery women who have no history of depression experience postpartum depression (1). If left untreated, this condition can disrupt the bonding between mother and child and create difficulties within the family. While psychotherapy and antidepressant medication are commonly recommended as initial treatments, some women with severe postpartum depression do not respond well to these approaches (2). Additionally, antidepressant medication can lead to unnecessary adverse reactions. Given the harmful consequences associated with postpartum depression (3), there is a clear need to develop additional therapies to better manage this condition.

Xiaoyao powder/pill, also known as Free and Easy Wanderer Powder, is a well-known Chinese herbal remedy that has gained attention for its potential to alleviate mood disorders, including postpartum depression (4). This prescription is composed of a combination of herbal ingredients, including *Radix Paeoniae Alba*, *Radix Angelicae Sinensis*, *Herba Menthae, Radix Bupleuri*, White *Poria*, *Rhizoma Atractylodis Macrocephalae*, *Radix Glycyrrhizae Praeparata*, and *Rhizoma Zingiberis Recens*. According to Traditional Chinese Medicine (TCM), this combination of herbs is believed to regulate the liver, soothe emotional distress, and harmonize the balance of Qi and blood, which are central concepts in treating mood disorders. Emerging research suggests that Xiaoyao formula (XYF) may help alleviate depressive symptoms by regulating neurotransmitters (such as serotonin, dopamine, and gamma-aminobutyric acid), the HPA axis, synaptic plasticity, inflammation, brain-derived neurotrophic factor, and the brain-gut axis (5). While several randomized clinical trials (RCTs) (6–11) have shown promising results when XYF was used in conjunction with Western therapy to alleviate depressive symptoms in postpartum women. However, there remains a lack of robust evidence due to the small sample sizes in these studies.

A recently published systematic review with meta-analysis has demonstrated that modified Xiaoyao powder, when used alone or in conjunction with Western therapy, could effectively reduce depressive symptoms in postpartum depression (12). However, this well-designed study did not differentiate between the original and modified Xiaoyao powder. Notably, the application of modified Xiaoyao powder poses significant challenges for non-TCM practitioners. This meta-analysis aims to synthesize the available evidence from RCTs to evaluate the effectiveness of XYF as an adjuvant therapy for treating postpartum depression.

## Methods

### Search strategy

We followed the Preferred Reporting Items for Systematic Reviews and Meta-Analyses guideline (13) to report the current study. The study analyzed data from individual studies and did not require ethical approval. We searched for articles indexed in international databases (Cochrane Library, Embase, and PubMed) and Chinese databases (SinoMed, CNKI, and Wanfang) from their inception to August 12, 2024, using the following terms (**Supplementary Text S1**): (“xiao yao” OR “Xiaoyao” OR “Shiau-Yau San”) AND (“postpartum depression” OR “postnatal depression”) AND (“controlled”) AND (“random”). We also manually reviewed the reference lists of the eligible RCTs for any potentially missing trials.

### Inclusion and exclusion criteria

Eligible studies met the following criteria: 1) Participants: women diagnosed with postpartum depression (based on the Hamilton Depression Scale [HAMD] or other valid international depression scales); 2) Intervention: XYF in combination with Western therapy; 3) Comparison: Western therapy alone; 4) Outcomes: total response rate, clinical recovery rate, and changes in postpartum depression scores; and 5) Study design: RCTs. The total response rate was defined as a reduction of more than 25% in depression scores, while a clinical recovery rate was defined as a reduction of more than 75% in depression scores. The exclusion criteria were as follows: 1) modified XYF or XYF combined with other complementary therapies as an intervention; 2) response rate defined by reducing depressive symptoms rather than a quantitative reduction in depression score; and 3) retrospective studies or self-controlled trials. Two independent reviewers (Liu J and Rong AN) screened all relevant studies based on predefined inclusion criteria. Disagreements between reviewers were resolved through discussion or consultation with a third reviewer (Wang F).

### Data extraction and risk of bias assessment

Two authors independently extracted the following information from the included studies: first author’s name, publication year, number of delivery women, age, criteria for diagnosis of postpartum depression, preparations and dosages of XYF used, types of antidepressants, length of treatment, and endpoints. The Cochrane risk of bias tool for RCTs was employed to assess the study quality. This tool evaluated the generation of random sequences, allocation concealment, blinding method, selective reporting of outcomes, and other sources of bias (such as selection of delivery women based on TCM syndrome). For data extraction and risk of bias assessment, the same set of reviewers followed a pre-designed, standardized data extraction form. Any discrepancies were identified by cross-checking and resolved through group discussion.

### Data synthesis and analysis

All meta-analyses were conducted using STATA version 12.0 (Stata Corp LP, College Station) and Review Manager version 5.1. The risk ratio (RR) with a 95% confidence interval (CI) was used to combine dichotomous data, while the standard mean difference (SMD) with a 95% CI was used for continuous data. Significant heterogeneity among the included trials was defined as a *p*-value of ≤ 0.05 for the Cochrane Q test and an *I* ^2^ statistic >50%. A random-effects or fixed-effects model was chosen based on the presence or absence of significant heterogeneity. A leave-one-out sensitivity analysis was performed to recalculate the pooled effect sizes. Subgroup analyses were conducted based on sample sizes, preparations of XYF, length of therapy, type of Western therapy, and whether patients were selected based on TCM syndrome differentiation. To determine publication bias, we used the Begg’s test and Egger’s test. If publication bias was detected, a trim-and-fill analysis was employed to adjust the pooled effect size.

## Results

### Search results and study characteristics

**Figure 1:**
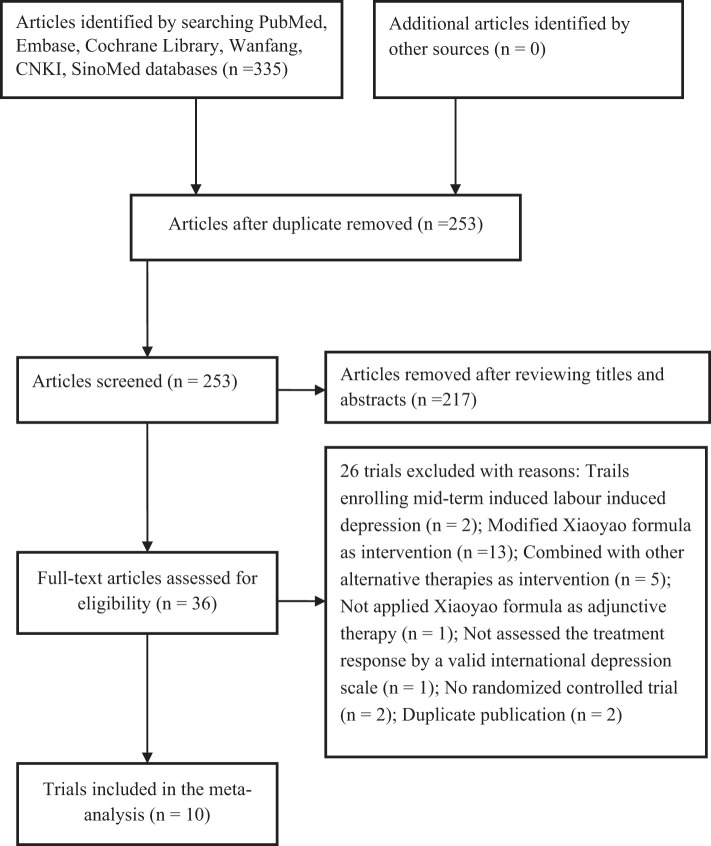
Flow chart of trial selection process.

Initially, we obtained 335 publications using the search strategy. Of these, 82 duplicate records were excluded, leaving 253 articles for evaluation of the titles and abstracts. After scanning the titles and abstracts, we retrieved 36 articles for full-text evaluation. Based on the criteria for inclusion and exclusion, 10 RCTs (6–11, 14–17) were ultimately included in this meta-analysis (**Figure 1**)

**Table 1** presents the main features of the included RCTs. All the RCTs were carried out in China and were published between 2008 and 2021. The sample sizes of the individual trials ranged from 64 to 120, with a total of 810 delivery women. Most of the RCTs used the Chinese Classification and Diagnostic Criteria of Mental Disorders-3 and the Diagnostic and Statistical Manual of Mental Disorders to diagnose depression. The XYF preparation included pills, powders, and decoctions. The included RCTs used various antidepressant medications, such as paroxetine, fluoxetine, sertraline, venlafaxine, escitalopram, amitriptyline, and deanxit. The study quality was summarized in **Supplementary Figures S1**, **S2**. Overall, all eligible RCTs were of suboptimal methodological quality with an unclear risk of bias.

**Table 1:** Baseline characteristics of trials included in meta-analysis.

First author/Year	Sample size	Mean age or age range	Diagnostic criteria	Experimental group	Control group	Treatment course	Outcome measures
Lin H 2008 (6)	Exp:37 Con:31	Exp: 27.1 ± 4.2 Con: 26.6 ± 4.2	DSM-IV HAMD≥20	XiaoYao Powder 18 g/d + Paroxetine 20 mg/d	Paroxetine 20 mg/d	6 weeks	Total response rate, clinical recovery rate, HAMD
Wei CL 2009 (7)	Exp:32 Con:32	Exp: 27.4 ± 5.1 Con: 26.7 ± 4.7	CCMD-3 HAMD≥17	XiaoYao Pill 24 pills/d + Sertraline 50-150 mg/d	Sertraline 50-150 mg/d	6 weeks	Total response rate, clinical recovery rate, HAMD
Liang QY 2012 (8)	Exp:60 Con:60	Exp:26.1 ± 4.2 Con: 25.7 ± 5.0	DSM-IV; HAMD≥14	XiaoYao Pill 24 pills/d + Fluoxetine 20mg/d	Fluoxetine 20 mg/d	6 weeks	HAMD
Wu Q 2014 (9)	Exp:35 Con:35	Exp: 23.7 ± 2.6 Con: 24.5 ± 2.7	CCMD-3 HAMD≥17	XiaoYao Powder + Sertraline	Sertraline	3 months	Total response rate, clinical recovery rate, HAMD
Li SY 2016 (10)	Exp:32 Con:32	Exp: 27.6 ± 5.2 Con: 26.8 ± 4.6	CCMD-3 HAMD≥17	XiaoYao Pill 24 pills/d + Escitalopram 10-20 mg/d	Escitalopram 10-20 mg/d	6 weeks	Clinical recovery rate, HAMD
Ma XL 2019 (11)	Exp:39 Con:37	Exp: 26.9 ± 2.1 Con: 26.5 ± 2.3	CCMD-3 HAMD≥17	XiaoYao Pill 18 g/d + Venlafaxine 75-225 mg/d	Venlafaxine 75-225 mg/d	6 weeks	Total response rate, clinical recovery rate, HAMD
Lin X 2020 (14)	Exp:36 Con:35	Exp: 27.0 ± 2.3 Con: 27.6 ± 2.1	CCMD-3 HAMD≥17	XiaoYao Pill 18 g/d + Paroxetine 10-40 mg/d	Paroxetine 10-40 mg/d	6 weeks	HAMD
Luo ZN 2020 (15)	Exp:35 Con:35	Exp: 27.5 ± 2.2 Con: 27.8 ± 1.9	Not reported	XiaoYao Decoction + Fluoxetine 20 mg/d	Fluoxetine 20 mg/d	4 weeks	SDS
Deng JX 2021 (16)	Exp:52 Con:52	Exp: 19-38 Con: 20-37	CCMD-3 HAMD≥17	XiaoYao Pill 24 pills/d + Deanxi 10-20 mg/d	Deanxit 10-20 mg/d	6 weeks	Total response rate, clinical recovery rate, HAMD
Zhang X 2021 (17)	Exp:53 Con:53	Exp: 28.3 ± 4.75 Con: 28.3 ± 4.52	Not reported	XiaoYao Decoction + Amitriptyline 25-50 mg/d	Amitriptyline 25-50 mg/d	8 weeks	SDS

CCMD, Chinese Classification of Mental Disorders; DSM, Diagnostic and Statistical Manual of Mental Disorders; HAMD, Hamilton Depression Scale; SDS, self-rating depressive scale.

### Total response rate

**Figure 2:**
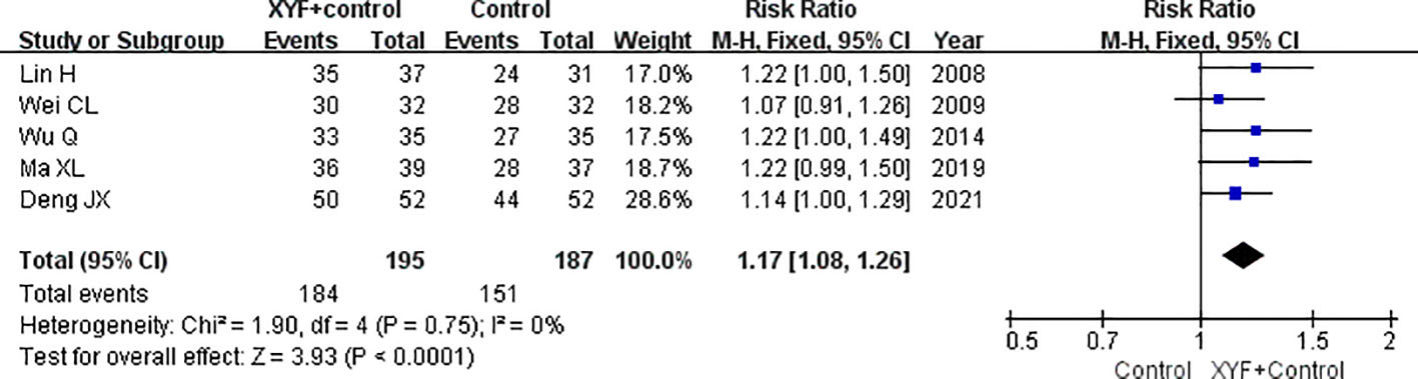
The pooled RR with a 95% CI for the total response rate of the XYF combined with Western therapy compared to Western therapy alone. XYF =Xiaoyao formula.

Five trials (6, 7, 9, 11, 16) examined the effect of XYF on the total response rate, defined as a reduction of more than 25% in depression scores. As shown in **Figure 2**, a fixed-effect model was used due to the lack of significant heterogeneity (*I*² = 0.0%; *p* = 0.750). The pooled RR for the total response rate was 1.17 (95% CI 1.08-1.26) for the combination of XYF and Western therapy compared to Western therapy alone. A sensitivity analysis, in which one trial was removed at a time, confirmed that no individual trial significantly influenced the overall risk estimate.

### Clinical recovery rate

**Figure 3:**
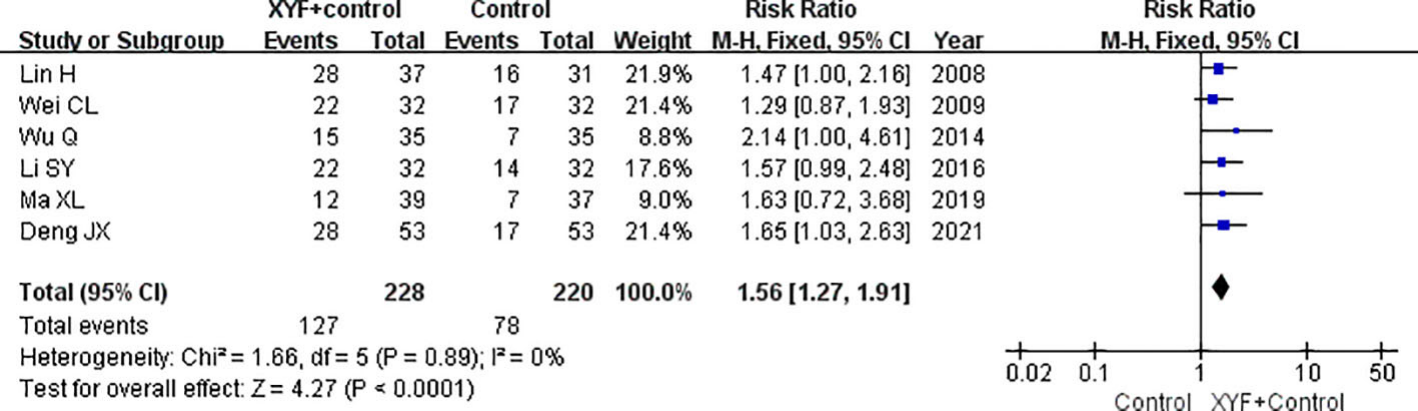
The pooled RR with a 95% CI for the clinical recovery rate of the XYF combined with Western therapy compared to Western therapy alone. XYF =Xiaoyao formula.

Six trials (6, 7, 9–11, 16) examined the effect of XYF on the total response rate, defined as a reduction of more than 75% in depression scores. As shown in **Figure 3**, a fixed-effect model was used due to the lack of significant heterogeneity (*I* ^2^ = 0.0%; *p*=0.890). The combined RR for the clinical recovery rate was 1.56 (95% CI 1.27-1.91) for the combination of XYF and Western therapy compared to Western therapy alone. None of the individual trials had a significant impact on the overall risk estimate in the sensitivity analysis.

### Improvement in depression scores

**Figure 4:**
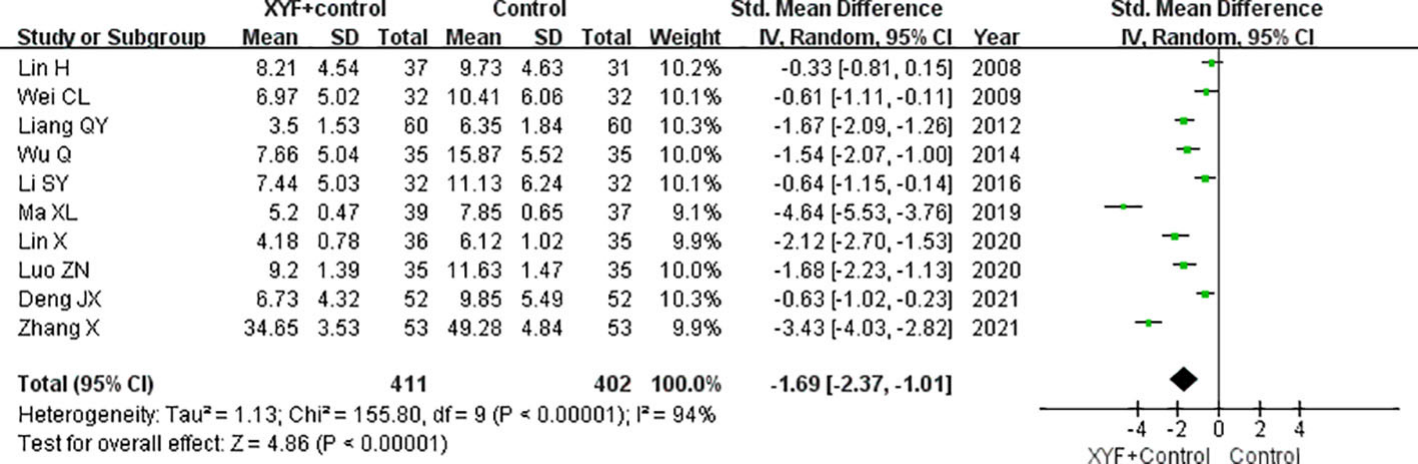
The pooled SMD with a 95% CI for the improvement in depression scores of the XYF combined with Western therapy compared to Western therapy alone. XYF =Xiaoyao formula.

All the trials included provided data on changes in depression scores following treatment. **Figure 4** shows significant heterogeneity among the trials (*I* ^2^ = 94.0%; *p*<0.001). A meta-analysis utilizing a random effects model found that the combination of XYF and Western therapy significantly reduced depression scores (SMD -1.69; 95% CI -2.37 to -1.01) compared to Western therapy alone. A leave-one-out sensitivity analysis further confirmed the reliability of the original pooled effect sizes (**Supplementary Figure S3**). Subgroup analyses consistently revealed significant effects of XYF on improving depression scores across each predefined subgroup (**Table 2**). Begg’s test (*p* = 0.004) and Egger’s test (*p* = 0.012) suggested a likelihood of publication bias. However, the “trim-and-fill” analysis indicated that the corrected effect size was only slightly underestimated (SMD -1.85; 95% CI -2.57 to -1.13).

**Table 2:** Subgroup analysis on reduction in depression scale.

Subgroups	Number of studies	Pooled SMD	95% CI	Heterogeneity between studies
Sample sizes >100 ≤ 100	3 7	-1.89 -1.61	-3.33 to -0.45 -2.44 to -0.77	p<0.001; I2 = 96.6% p<0.001; I2 = 93.7%
Form of Xiaoyao formula Pill Others	6 4	-1.66 -1.73	-2.55 to -0.78 -2.96 to -0.51	p<0.001; I2 = 94.5% p<0.001; I2 = 95.2%
Course of treatment >6 weeks ≤6 weeks	2 8	-2.48 -1.49	-4.33 to -0.62 -2.19 to -0.79	p<0.001; I2 = 95.2% p<0.001; I2 = 93.4%
Depression scale HAMD Others	8 2	-1.47 -2.55	-2.16 to -0.77 -4.26 to -0.84	p<0.001; I2 = 93.3% p<0.001; I2 = 94.3%
Type of antidepressant SSRI Others	7 3	-1.22 -2.88	-1.72 to -0.72 -5.34 to -0.42	p<0.001; I2 = 85.5% p<0.001; I2 = 98.0%
TCM syndrome differentiation Yes No	5 5	-1.76 -1.63	-2.75 to -0.76 -2.70 to -0.56	p<0.001; I2 = 94.8% p<0.001; I2 = 94.8%

SMD, standard mean difference; CI, confidence interval; HAMD, Hamilton Depression Scale; SSRI, selective serotonin reuptake inhibitor

## Discussion

This study represents the first meta-analysis to evaluate the effectiveness of XYF as an adjunctive therapy for postpartum depression. Our primary findings show that using XYF in addition to Western therapy leads to a significant improvement in both the total response rate (17%) and clinical recovery rate (56%) compared to using Western therapy alone. Furthermore, the adjuvant treatment with XYF led to a substantial reduction in depression (SMD -1.69) scores. This large effect size indicates that XYF has a clinically meaningful impact on reducing depressive symptoms. These findings suggest that combining XYF with Western therapy can greatly enhance its anti-depressive effectiveness.

There is a growing body of evidence supporting the beneficial effects of Xiaoyao powder/pill in treating depression. Previous meta-analyses (18–20) have demonstrate that XYF can effectively alleviate depressive symptoms in patients with depressive disorder. Furthermore, a recent meta-analysis (21) concluded that XYF, when used as an adjuvant therapy, provided further benefits by reducing HAMD scores in patients with post-stroke depression. However, these previous meta-analyses did not include patients with postpartum depression. Therefore, the current meta-analysis specifically evaluated the XYF as an adjunctive therapy in recently delivery women diagnosed with depression.

The precise mechanisms underlying the beneficial effects of XYS in the treatment of postpartum depression remain largely unclear. However, research has shown that changes in the levels of estradiol and monoamine neurotransmitters may play a role in the onset of postpartum depression (22). For example, a study has found that women with postpartum depression have lower levels of estradiol and 5-hydroxytryptamine in their blood compared to healthy women (23). Furthermore, adjuvant treatment with XYF has been shown to increase levels of estradiol, noradrenaline, dopamine, and 5-hydroxytryptamine in the blood (11, 14).

XYF, when used as an adjuvant therapy, demonstrated additional beneficial effects in postpartum depression. Syndrome differentiation is a crucial aspect of TCM; however, only five RCTs (7–9, 11, 16) considered this during the patient selection process. XYF is particularly suitable for treating depressive symptoms induced by liver–stomach disharmony syndrome. Our subgroup analysis further supports the superiority of RCTs that incorporate syndrome differentiation, as evidenced by a greater reduction in depression scale scores (SMD 1.76) compared to those that do not (SMD 1.63).

However, there was significant heterogeneity in the pooled depression scores when treated as continuous variables. This indicates considerable variation among the studies, which weakened the strength of our overall conclusion. To address this issue, we conducted a leave-one-out sensitivity analysis. This analysis demonstrated that our overall conclusion remained stable, suggesting that no single study was responsible for the observed heterogeneity. When categorizing the studies based on the type of antidepressant, we found that heterogeneity was reduced within the subgroups of selective serotonin reuptake inhibitors. This implies that the type of antidepressant may be a partial contributor to the heterogeneity. However, the method used to assess depression is a significant factor contributing to this variability. Additionally, the severity of depression among participants and variations in treatment regimens may also play a role in the observed heterogeneity.

Our meta-analysis results indicate that XYF is a promising adjunctive therapy for postpartum depression, demonstrating significant improvements in response rates, recovery rates, and depression scores. Sertraline belongs to a class of drugs known as selective serotonin reuptake inhibitors, which has been recommended as one of the safest antidepressants during breastfeeding in the context of balancing risks and benefits (24). Healthcare providers should consider incorporating XYF into treatment plans for postpartum depression, especially for patients who show inadequate response to Western therapy alone. As an adjunctive therapy, XYF can enhance the overall efficacy of conventional treatments, potentially reducing the need for higher doses of antidepressants or other medications that may carry side effects. Furthermore, patient selection based on TCM syndrome differentiation may further optimize the therapeutic benefits of XYF.

The current meta-analysis shows that XYF has short-term benefits for depression, but long-term RCTs are necessary to assess its sustained effects and safety during extended use. Future research should focus on incorporating pharmacogenetic testing to tailor treatment plans more effectively, taking into account the unique genetic profiles of individuals. Additionally, studies should compare XYF with other complementary and alternative therapies to determine its relative efficacy. Head-to-head trials comparing XYF with conventional antidepressants could also provide valuable insights into its potential as a first-line or adjunctive treatment. Further preclinical and clinical studies are needed to fully understand the mechanisms by which XYF alleviates depression.

We must acknowledge several limitations in our meta-analysis. Firstly, the lack of detailed descriptions of allocation concealment and blinding methods makes it difficult to assess the quality of the studies and the potential for bias. Secondly, the lack of clarity in the patient selection process could result in the inclusion of patients who do not have the specific TCM syndrome that XYF is intended to treat, potentially skewing the results. Thirdly, the sample sizes and the number of included trials were relatively small, necessitating caution when interpreting the results of subgroup analyses. Fourthly, the long-term efficacy of XYF for postpartum depression remains uncertain due to a lack of follow-up information. Finally, we did not conduct a quantitative analysis of adverse events associated with XYF, as the indices of adverse events were reported in various formats. However, no severe adverse events were documented in any of the included trials.

## Conclusions

XYF as an adjunctive therapy offers additional benefits in alleviating depression in postpartum women. However, the current evidence remains uncertain due to the methodological flaw of the analyzed trials. Future research should focus on large, multi-center RCTs, dose-response studies, head-to-head comparisons with Western therapy, and investigations into XYF's mechanisms of action.

## Data Availability

The original contributions presented in the study are included in the article/**Supplementary Material**. Further inquiries can be directed to the corresponding author.
