# Supplementary material for: Meta-analysis of Xiaoyao formula as an adjuvant therapy for treating postpartum depression
Source: Front Psychiatry. 2025 Mar 24;16:1558505. doi: 10.3389/fpsyt.2025.1558505 (PMC11973275; doi:10.3389/fpsyt.2025.1558505)
Supplement: Supplementary file 1 [file SupplementaryFile1.docx]

**Supplementary Text S1 Search strategy**

| **1. PubMed** | Query | | Items found |
| --- | --- | --- | --- |
| #1 | Search: (((postpartum depression) OR (postnatal depression)) OR (post-natal depression))) | | 19,652 |
| #2 | Search: **(((xiaoyao) OR (xiao yao)) OR (Shiau-Yau San)) OR (Shoyo San)** | | 1,260 |
| #3 | Search: **(#1) AND (#2)** | | **3** |
|  |  | |  |
|  |  | |  |
| **2. Embase** | Query | Items found | |
| #1 | Search 'postpartum depression'/exp OR 'postpartum depression' OR (('postpartum'/exp OR postpartum) AND ('depression'/exp OR depression)) | 23,756 | |
| #2 | Search post-partum depression'/exp OR 'post-partum depression' OR ('post partum' AND ('depression'/exp OR depression)) | 17,849 | |
| #3 | Search 'postnatal depression'/exp OR 'postnatal depression' OR (postnatal AND ('depression'/exp OR depression)) | 24,526 | |
| #4 | Search 'post-natal depression'/exp OR 'post-natal depression' OR ('post natal' AND ('depression'/exp OR depression)) | 17,373 | |
| #5 | Search #1 OR #2 OR #3 OR #4 | 35,235 | |
| #6 | Search 'xiaoyao'/exp OR xiaoyao | 892 | |
| #7 | Search 'xiao yao' OR (xiao AND yao) | 22,194 | |
| #8 | Search 'shiau-yau san' OR ('shiau yau' AND san) | 6 | |
| #9 | 'shoyo san' OR (shoyo AND san) | 27 | |
| #10 | Search #6 OR #7 OR #8 OR #9 | 23,011 | |
| #11 | Search #5 AND #10 | **9** | |

**3. Cochrane Library**

## Search: “(xiaoyao powder or xiao yao san)” OR “(xiaoyao pill or xiao yao wan)” OR “Shiau-Yau San” OR “Shoyo San” in All Text 115 items

**4.** **China National Knowledge Infrastructure**

Search (产后抑郁[Title]) AND (逍遥[All text]) AND (随机[All text]) AND (对照[All text]) **127 items**

**5. Chinese Biomedical Literature Database (Sinomed)**

Search ("产后抑郁"[Title]) AND "逍遥"[ All fields] AND "随机"[ All fields] AND "对照"[ All fields] **40 items**

**6. Wanfang Database**

Search (((逍遥) AND 产后抑郁) AND 随机) AND 对照 **41 Items**
