# Supplementary material for: Meta-analysis of Xiaoyao formula as an adjuvant therapy for treating postpartum depression
Source: Front Psychiatry. 2025 Mar 24;16:1558505. doi: 10.3389/fpsyt.2025.1558505 (PMC11973275; doi:10.3389/fpsyt.2025.1558505)

Supplemental Figure S1 Risk of bias graph


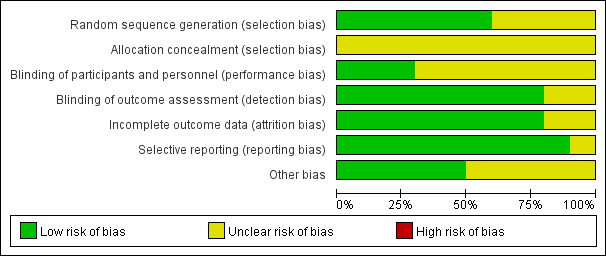


Supplemental Figure S2 Risk of bias summary


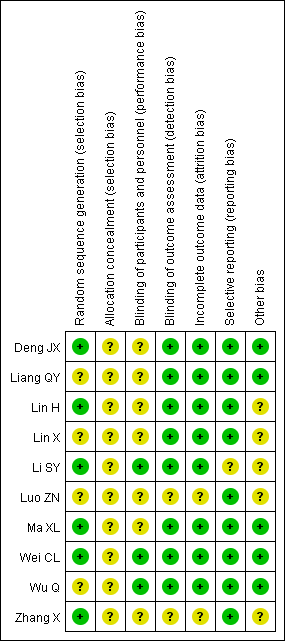


Supplemental Figure S3 Results of leave-one-out sensitivity analysis


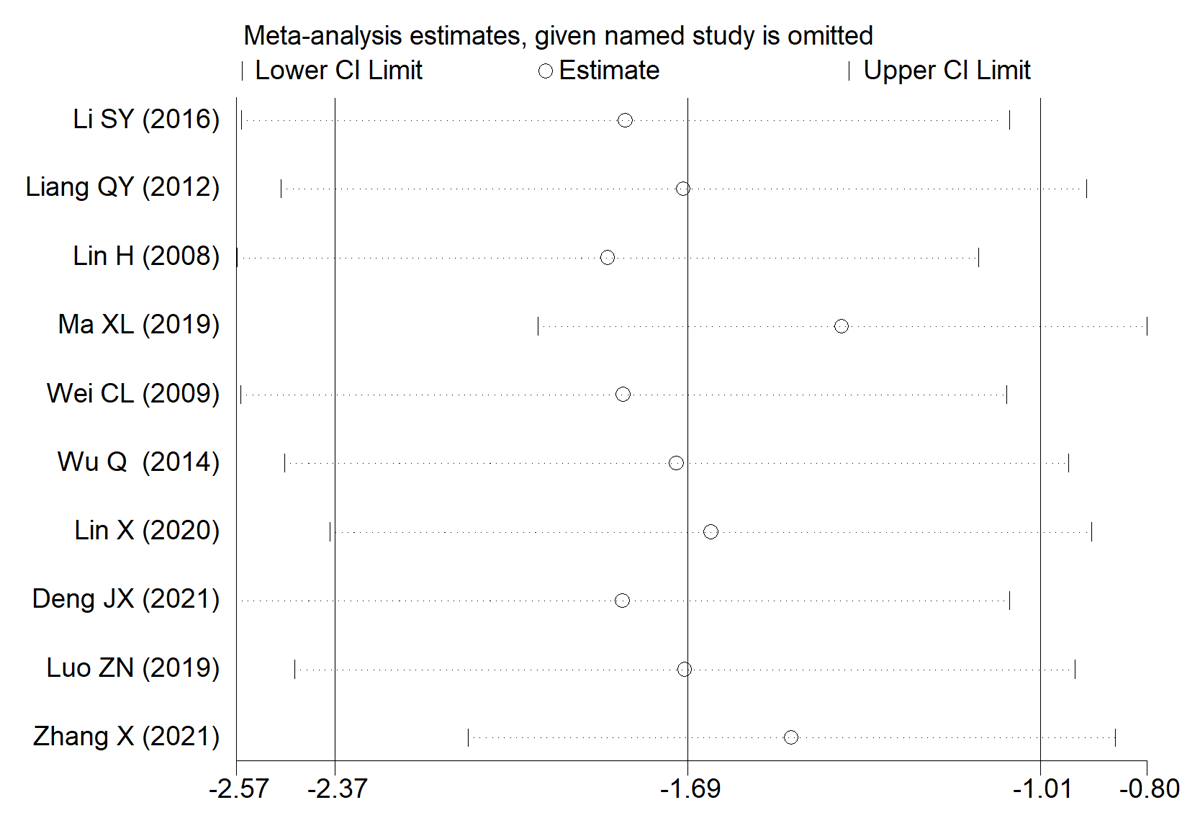

Supplement: Supplementary file 2 [file SupplementaryFile2.docx]
